# A cross-sectional survey of Chinese anesthesiologists’ understanding of fluid resuscitation recommendations in the 2021 surviving sepsis campaign guidelines

**DOI:** 10.1186/s12871-026-03990-x

**Published:** 2026-06-05

**Authors:** Wenli Zhao, Jiangchen Zhao, Jinhua Zhang, Ping Chen, Hui Li

**Affiliations:** 1 Department of Anesthesiology, The First Affiliated Hospital, School of Medicine, Zhejiang University, Hangzhou, China; 2 Department of Anesthesiology, The First People’s Hospital of Yuhang District, Hangzhou, China

**Keywords:** Surviving sepsis campaign guidelines, Sepsis, Fluid resuscitation, Anesthesiologists, Self-reported adherence

## Abstract

**Background:**

This study evaluated whether anesthesiologists from various backgrounds are familiar with the 2021 Surviving Sepsis Campaign (SSC) fluid resuscitation recommendations and identified areas for targeted education and practice improvements.

**Methods:**

The internet-based survey was conducted from March 13 to April 13, 2024, using Tencent Questionnaire via WeChat. Data concerning respondents’ demographics, self-reported familiarity with and adherence to SSC fluid resuscitation recommendations, and specific knowledge questions were collected. Comparisons were made between anesthesiologists from hospitals across different tiers to evaluate differences in understanding. Multivariable logistic regression was performed to adjust for potential confounders.

**Results:**

In total, 1,227 anesthesiologists completed the survey; after excluding non-anesthesiologists, 1,051 were included in the analysis. Among them, 7.2% reported being “very familiar” and 28.4% reported being “somewhat familiar” with fluid resuscitation strategies outlined in the SSC guidelines. Moreover, 72.2% of anesthesiologists reported following the SSC guidelines during perioperative management of septic patients. Although self-reported familiarity and adherence did not significantly differ across hospital tiers, differences emerged in specific areas of knowledge, including the choice of first-line fluid for resuscitation and the selection of first-line vasopressor. However, after adjusting for years of clinical experience, professional rank, academic degree, and annual sepsis case volume, hospital tier was no longer significantly associated with knowledge of either first-line fluid or first-line vasopressor selection.

**Conclusions:**

This nationwide cross-sectional survey suggests that while more than half of Chinese anesthesiologists reported general familiarity with and adherence to the SSC guidelines, many lacked updated knowledge of specific 2021 recommendations. Individual-level factors were associated with guideline familiarity and knowledge, whereas institutional characteristics appeared to play a limited role after adjustment. These findings may help identify gaps and inform targeted educational interventions for junior anesthesiologists and those with lower academic qualifications, though causal inferences are precluded by the observational design.

**Supplementary Information:**

The online version contains supplementary material available at 10.1186/s12871-026-03990-x.

## Introduction

Sepsis is defined as life-threatening organ dysfunction due to a dysregulated host response to infection [1]. Approximately 20–30% of ICU admissions are due to sepsis or septic shock, and 25% to 40% of these patients die before hospital discharge [2]. The Surviving Sepsis Campaign (SSC), initially aimed to reduce sepsis-related mortality by 25% within 5 years [3], has since published the 5th Edition of International Guidelines for the Management of Sepsis and Septic Shock, based on the latest evidence [4]. However, adherence to these guidelines remains suboptimal, particularly among non-intensive care specialists. A U.S. study found that only 42.5% of emergency physicians and 47.4% of internists selected norepinephrine as first-line vasopressor, compared to 72.5% of intensivists [5]. Similarly, a multicenter survey revealed that < 20% of internal and emergency medicine house staff correctly identified diagnostic criteria for sepsis [6]. These findings underscore the need to assess guideline awareness among anesthesiologists, who frequently initiate resuscitation in the perioperative setting.

In China, the healthcare system is hierarchically organized into tertiary (including grade 3 A, 3B and 3 C), secondary, and primary hospitals based on bed capacity, technical capabilities, and service scope. Anesthesiologists practice across all tiers, often assuming dual roles as perioperative physicians and, in smaller institutions, direct participants in critical care management. This organizational context creates heterogeneous exposure to sepsis care protocols among anesthesia providers. Timely updates to their knowledge of sepsis diagnosis and treatment, can substantially improve patient outcomes and poor compliance has been associated with higher sepsis-related mortality [7, 8]. No study has specifically assessed Chinese anesthesiologists’ familiarity with the 2021 SSC guideline updates, particularly regarding revised fluid resuscitation recommendations. This study aims to fill this gap and pinpoint knowledge deficits that could guide targeted education and quality-improvement initiatives.

## Methods

### Survey design and population

This questionnaire-based, cross-sectional study was reported in accordance with the STROBE (Strengthening the Reporting of Observational Studies in Epidemiology) guideline for cross-sectional studies. The completed STROBE checklist is provided as Supplementary Material 1.

The study recruited anesthesiologists from hospitals across different tiers in China who participated on a voluntary basis. The survey was open from March 13 to April 13, 2024. No incentives to complete the survey were offered.

The questionnaire was developed by the authors specifically for this study, based on the 2021 SSC guidelines, and has not been previously published. It was pilot-tested with 10 anesthesiologists from a tertiary hospital to evaluate face validity, clarity, and completion time. The questionnaire, provided as Supplementary Material 2, consists of 33 items divided into two sections, with an estimated completion time of 5–15 min.

(1) demographic and career data (geographic region, years of clinical experience, professional rank, academic degree, hospital category/size, and annual sepsis case volume); and (2) self-reported familiarity with and adherence to SSC fluid resuscitation recommendations (fluid therapy strategies, fluid selection, resuscitation markers, and influencing factors) and specific knowledge questions (target mean arterial pressure (MAP) and first-line vasopressors). Familiarity was assessed using a four-point scale: “very familiar” (confident in applying the recommendations), “somewhat familiar” (basic understanding but may need to reference guidelines), “general knowledge” (aware of the recommendations but not confident in application), and “don’t know” (no prior knowledge).

Inclusion criteria: practicing anesthesiologists with complete responses to all core questions. Exclusion criteria: non-anesthesiologist status or > 50% missing data.

### Questionnaire distribution

The questionnaire was created using Tencent Questionnaire, a widely used online survey platform in China. For distribution, the research team initially posted the survey link on WeChat, and then the link was shared across various anesthesiology-focused WeChat groups (including society-affiliated, departmental, and continuing medical education groups). Recipients were encouraged to forward the questionnaire to other eligible anesthesiologists, employing a snowball sampling strategy.

Data collection lasted from March 13 to April 13. The platform’s settings prevented multiple submissions from the same IP address to minimize duplicate responses. Upon closure of the survey, data were exported directly from the Tencent Questionnaire platform into Microsoft Excel for cleaning and subsequent statistical analysis. Due to the snowball sampling approach, the exact number of unique individuals reached and the number of non-respondents could not be determined. Therefore, traditional response rate calculation was not feasible. However, the Tencent Questionnaire platform provided a function to track the number of unique visitors who opened the link.

### Non-response bias assessment

To assess non-response bias, we compared early respondents (first 20%, *n* = 210) with late respondents (last 20%, *n* = 210). No significant differences were observed in years of experience (*P* = 0.467) or professional rank (*P* = 0.234). However, early respondents were more likely to work in university-affiliated tertiary hospitals (68.6% vs. 51.0%), while late respondents were more likely to work in primary hospitals (21.4% vs. 5.7%, *P* < 0.001). To address this imbalance, we conducted stratified analyses by hospital tier and found no significant differences between early and late respondents within each tier regarding guideline adherence, familiarity, or knowledge (all *P* > 0.05). These findings suggest that although response patterns varied by hospital level, the overall bias direction was consistent across tiers, supporting the robustness of our main results.

### Statistical analysis

Statistical analysis was performed utilizing SPSS version 25.0. Counts and percentages summarized categorical variables. Comparisons of categorical and non-continuous variables between groups were conducted using the χ^2^ test or Fisher’s exact test, as appropriate. Bonferroni correction was applied for multiple comparisons across hospital tiers.

Multivariable logistic regression was performed to assess the independent association between hospital tier and key outcomes, adjusting for potential confounders: years of clinical experience, professional rank, academic degree and self-reported annual sepsis case volume. Adjusted odds ratios (a OR) with 95% confidence intervals (CI) were reported. P-value < 0.05 was considered statistically significant. To assess potential multicollinearity among the variables years of clinical experience, professional rank, and academic degree, we calculated variance inflation factors (VIF) analysis and it showed all values below 2.5, suggesting no severe multicollinearity requiring variable exclusion.

## Results

### Basic characteristics

During the data collection period from March 13 to April 13, the survey link was opened by 4936 unique visitors (based on Tencent Questionnaire platform analytics). A total of 1227 questionnaires were returned, yielding a completion rate of 24.9%. Of these, 1,051 (85.7%) met inclusion criteria and were analyzed. Exclusions were due to non-anesthesiologist status (*n* = 176). Among the 1,051 respondents, 604 (57.5%) worked in university-affiliated tertiary hospitals, 157 (14.9%) in non-affiliated tertiary hospitals, 118 (11.2%) in other tertiary hospitals, and 172 (16.4%) in primary hospitals.

### Self-reported familiarity and adherence to SSC fluid resuscitation recommendations

Of the 1,051 respondents, 7.2% reported being “very familiar” and 28.4% “somewhat familiar” with the fluid therapy strategies in the SSC guidelines; 50.4% had “general knowledge”, and 14% were unaware of these specific recommendations. In terms of self-reported adherence, 72.2% reported following the SSC guidelines for fluid management in septic patients during the perioperative period, while 27.8% did not. Regarding fluid therapy strategies, 45.4% of respondents selected the SSC-recommended approach (immediate fluid resuscitation followed by dynamic monitoring and reassessment). An additional 21.1% chose initial fluid resuscitation followed by physical examination or static reassessment. Notably, 33.5% selected early goal-directed therapy (EGDT), protocolized fluid resuscitation with specific hemodynamic targets (central venous pressure 8–12 mmHg, MAP ≥ 65 mmHg) within the first 6 h, a strategy that is no longer recommended in the 2021 guidelines.

### Perceived and actual knowledge of guidelines among familiar respondents

Because self-reported familiarity may be subject to bias, we assessed objective knowledge among respondents who reported being “very familiar” or “somewhat familiar” with the guidelines. Among the 374 such respondents (35.6%), we found notable discordance between perceived and actual knowledge: 10.5% (*n* = 39) did not select balanced crystalloids as first-line fluid. Among these, 87.2% cited “fluid availability” and 74.4% “department convention” as influencing factors. 36.1% (*n* = 135) did not identify 65 mmHg as the recommended target MAP. 8.8% (*n* = 33) did not select norepinephrine as first-line vasopressor.

### Knowledge of the SSC guidelines

For initial fluid resuscitation and subsequent volume replacement, the SSC guidelines recommend balanced crystalloids rather than normal saline; 84.6% of respondents selected this option. Regarding hydroxyethyl starch, which the guidelines explicitly advise against, 68.7% answered in accordance with the recommendation. For fluid resuscitation in sepsis or septic shock, the guidelines suggest adding albumin when substantial crystalloids are required; 52.5% selected this option.

Of the 1,051 respondents, 465 (44.2%) selected that patients with septic shock or strong suspicion of sepsis should receive broad-spectrum antibiotics within 1 h. For recommended hemodynamic targets, response rates were: MAP 53.3%, lactate levels 44.3%, urine output 61.8%, pulse pressure variation (PPV) 32.3%, and stroke volume variation (SVV) 63.8%. Norepinephrine was selected as the first-line vasopressor by 86.4%; adrenaline and dopamine were selected by 6.4% and 3.9%, respectively.

### Daily practice and influencing factors

In questions about daily clinical practice, 77.4% of anesthesiologists reported using MAP as the most common indicator to guide fluid therapy for patients with septic shock. This was followed by urine output (74.1%) and lactate levels (60%). Notably, only 6% of respondents selected capillary refill time (CRT), despite its recommendation in the latest guidelines as a supplemental perfusion monitoring method during resuscitation. When asked about factors influencing their clinical decisions, 81.5% of anesthesiologists cited adherence to guidelines, 75.5% reported limited access to routine tests within their hospital, and 68% noted challenges in conducting specific investigations.

The most preferred fluid for both elective surgeries and septic shock was lactated Ringer’s solution, chosen by 88% and 67.6% of anesthesiologists, respectively. For elective surgeries, the second most preferred fluid was hydroxyethyl starches, selected by 57.9% of respondents; albumin was the second choice for septic shock, selected by 50.6%. Acetated Ringer’s solution ranked third, selected by 39.3% of anesthesiologists for elective surgeries and 46.4% of anesthesiologists for septic shock. The three factors most strongly influencing fluid selection were fluid availability, department convention and SSC recommendations.

### Differences across hospital tiers

**Fig. 1:**
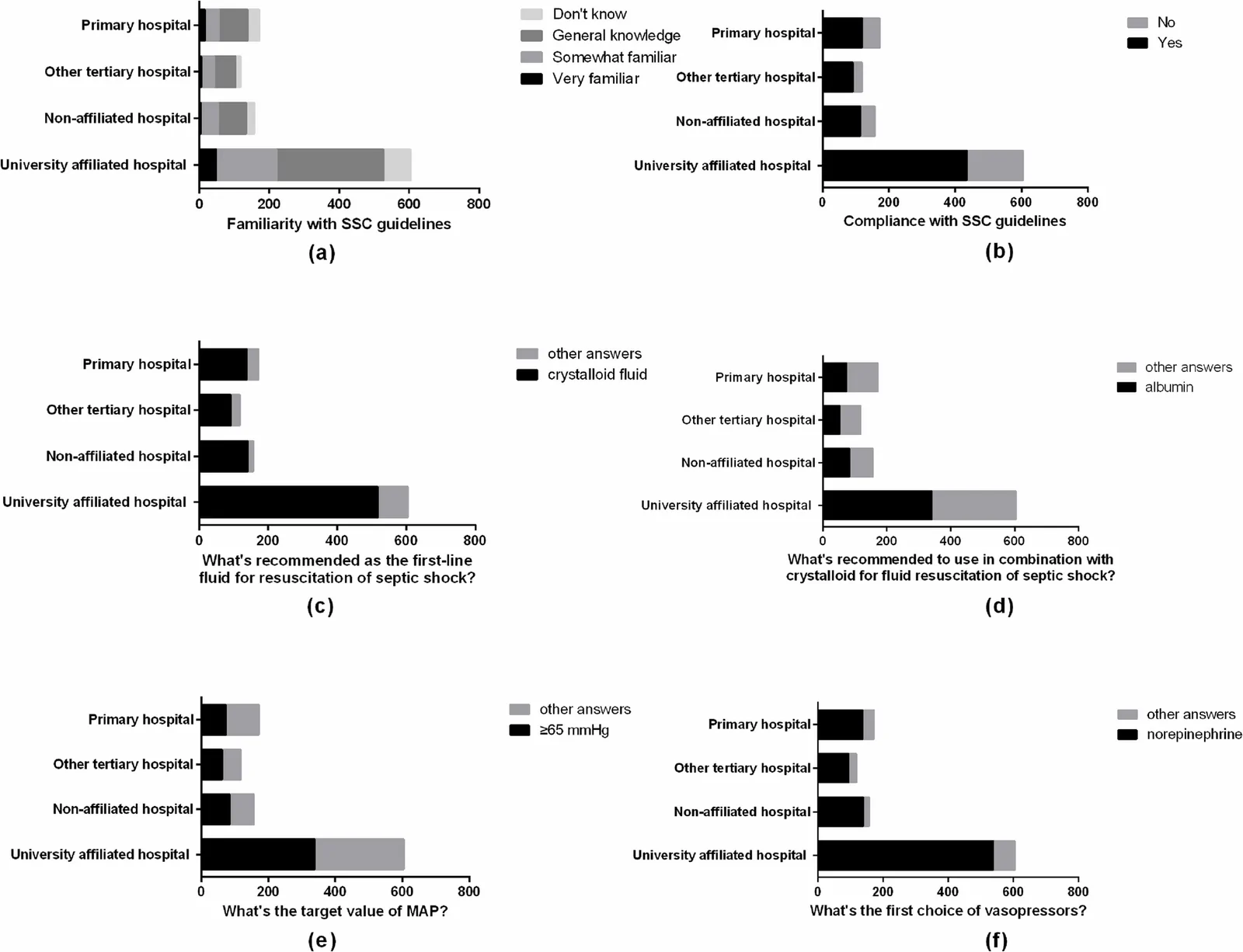
Comparison of anesthesiologists’ self-reported familiarity with or adherence to SSC fluid resuscitation recommendations across hospital tiers. SSC, Surviving Sepsis Campaign; MAP, mean arterial pressure. **a** Familiarity with SSC guidelines (*P* > 0.05). (b) Compliance with SSC guidelines (*P* > 0.05). **c** The first-line fluid for resuscitation of septic shock (*P* < 0.05). **d** What’s recommended to use in combination with balanced crystalloid for fluid resuscitation of septic shock (*P* < 0.05). **e** The target value of MAP (*P* < 0.05). **f** The first-line vasopressor (*P* < 0.05)

There were no statistically significant differences in overall self-reported familiarity with or adherence to SSC fluid resuscitation recommendations across hospital tiers (*P* = 0.158 and *P* = 0.527, respectively). However, differences emerged in specific areas of knowledge. Anesthesiologists at university-affiliated tertiary hospitals showed higher concordance with guideline recommendations regarding first-line fluid selection (*P* = 0.016), fluids recommended for combination with crystalloids (*P* = 0.005), and fluids not recommended for resuscitation (*P* = 0.005). Similarly, university-affiliated tertiary hospital anesthesiologists were more likely to identify the recommended MAP target (*P* = 0.025), PPV target (*P* < 0.001), SVV target (*P* < 0.001), and first-line vasopressor (*P* = 0.001) (Table 1; Fig. 1).

**Table 1:** Comparison of anesthesiologists’ self-reported familiarity with or adherence to SSC fluid resuscitation recommendations across hospital tiers

	Tertiary hospital (class A)		Other tertiary hospital	Primary hospital	*P*
	University affiliated hospital	Non-affiliated hospital			
Familiarity with SSC guideline					= 0.158
Very familiar	49 (8.1%)	5 (3.2%)	6 (5.1%)	16 (9.3%)	
Somewhat familiar	172 (28.5%)	50 (31.8%)	36 (30.5%)	40 (23.3%)	
General knowledge	306 (50.7%)	79 (50.3%)	62 (52.5%)	83 (48.3%)	
Don’t know	77 (12.7%)	23 (14.6%)	14 (11.9%)	33 (19.2%)	
Adherence to SSC guidelines					= 0.527
Yes	435 (72.1%)	114 (72.6%)	91 (77.1%)	119 (69.2%)	
Whether fluid selection for septic shock differs from that used in elective surgery					= 0.213
Yes	540 (89.4%)	131 (83.4%)	104 (88.1%)	149 (86.6%)	
Fluid therapy strategy for septic shock					= 0.007^*^
Early goal-directed therapy	222 (36.8%)	52 (33.1%)	34 (28.8%)	44 (25.6%)	
Initial fluid resuscitation followed by physical examination or static reassessment	104 (17.2%)	37 (23.6%)	30 (25.4%)	51 (29.7%)	
Immediate fluid resuscitation followed by dynamic monitoring and reassessment	278 (46.0%)	68 (43.3%)	54 (45.8%)	77 (44.8%)	
The first-line fluid for resuscitation in septic shock					= 0.016^*^
Balanced crystalloid	518 (85.8%)	141 (89.8%)	92 (78.0%)	138 (80.2%)	
What’s not recommended for fluid resuscitation of septic shock					= 0.005^*^
Hydroxyethyl starch	417 (69.0%)	122 (77.7%)	81 (68.6%)	102 (59.3%)	
What’s recommended to use in combination with crystalloid for fluid resuscitation of septic shock					= 0.005^*^
Albumin	342 (56.6%)	83 (52.9%)	53 (44.9%)	74 (43.0%)	
The recommended target mean arterial pressure					= 0.025^*^
≥65 mmHg	339 (56.1%)	85 (54.1%)	62 (52.5%)	74 (43.0%)	
The recommended target lactate					= 0.388
≤ 2 mmol/L	273 (45.2%)	75 (47.8%)	45 (38.1%)	73 (42.4%)	
The recommended target urine output					= 0.324
≥ 0.5 ml/kg/h	382 (63.2%)	100 (63.7%)	65 (55.1%)	102 (59.3%)	
The recommended target PPV					< 0.001^*^
0–10%	217 (35.9%)	58 (36.9%)	25 (21.2%)	39 (22.7%)	
The recommended target SVV					< 0.001^*^
SVV < 13%	430 (71.2%)	109 (69.4%)	56 (47.5%)	76 (44.2%)	
The first-line vasopressor					= 0.001^*^
Norepinephrine	538 (89.1%)	139 (88.5%)	94 (79.7%)	137 (79.6%)	

*Represents significant difference across different hospital tiers

### Multivariable logistic regression analysis

We performed multivariable logistic regression models for key outcome, adjusting for hospital tier, years of clinical experience, professional rank, academic degree, and annual sepsis case volume. The results are presented in Table 2.

**Table 2:** Multivariable logistic regression analysis of factors associated with self-reported familiarity and knowledge of the SSC guideline

Predictor	Familiarity with SSC guideline		The first-line fluid		The first-line vasopressor	
	Adjusted OR (95% CI)	*P*	Adjusted OR (95% CI)	*P*	Adjusted OR (95% CI)	*P*
Hospital tier (ref: primary)		= 0.042^*^		= 0.157		= 0.386
University-affiliated tertiary	0.50 (0.30–0.83)	= 0.008^*^	1.24 (0.67–2.29)	= 0.499	1.50 (0.79–2.86)	= 0.216
Non-university tertiary	0.64 (0.37–1.09)	= 0.101	1.80 (0.88–3.67)	= 0.105	1.42 (0.71–2.85)	= 0.327
Other tertiary	0.87 (0.52–1.47)	= 0.607	0.79 (0.44–1.45)	= 0.451	0.89 (0.48–1.64)	= 0.711
Experience (ref: <3 years)		= 0.895		= 0.015^*^		= 0.218
> 10 years	1.08 (0.48–2.43)	= 0.853	1.06 (0.43–2.60)	= 0.905	2.02 (0.78–5.24)	= 0.150
5–10 years	0.94 (0.44–2.01)	= 0.863	1.22 (0.55–2.72)	= 0.623	2.16 (0.91–5.14)	= 0.081
3–5 years	1.09 (0.56–2.12)	= 0.799	3.45 (1.52–7.81)	= 0.003^*^	2.14 (0.99–4.63)	= 0.054
Professional rank (ref: junior)		= 0.006^*^		= 0.058		= 0.071
Senior	3.94 (1.81–8.55)	= 0.001^*^	2.86 (1.07–7.64)	= 0.037^*^	1.75 (0.62–4.99)	= 0.292
Associate senior	2.53 (1.23–5.18)	= 0.011^*^	2.39 (1.02–5.65)	= 0.046^*^	1.37 (0.56–3.38)	= 0.494
Intermediate	2.12 (1.15–3.92)	= 0.016^*^	1.37 (0.68–2.79)	= 0.380	0.78 (0.37–1.62)	= 0.496
Academic degree(ref: associate degree)		= 0.000^*^		= 0.256		= 0.016^*^
Doctoral degree	7.09 (2.92–17.22)	= 0.000^*^	0.87 (0.33–2.29)	= 0.775	1.03 (0.37–2.73)	= 0.959
Master’s degree	2.99 (1.32–6.74)	= 0.008^*^	1.53 (0.64–3.62)	= 0.336	2.49 (1.05–5.90)	= 0.038^*^
Bachelor’s degree	2.50 (1.17–5.33)	= 0.018^*^	1.28 (0.60–2.74)	= 0.522	1.78 (0.86–3.72)	= 0.123
Case volume (ref: <50)		= 0.001^*^		= 0.476		= 0.039^*^
> 300	2.40 (1.51–3.83)	= 0.000^*^	1.29 (0.71–2.35)	= 0.406	1.26 (0.66–2.39)	= 0.488
150–300	1.93 (1.22–3.07)	= 0.005^*^	1.36 (0.74–2.48)	= 0.322	3.50 (1.50–8.17)	= 0.004^*^
50–150	1.77 (1.24–2.51)	= 0.001^*^	1.41 (0.90–2.20)	= 0.132	1.17 (0.75–1.83)	= 0.500

*Represents significant group difference

For self-reported familiarity with the SSC guidelines, multivariable logistic regression analysis revealed multiple independent predictors, with professional rank, academic degree, and annual sepsis case volume demonstrating the strongest associations. In contrast, years of clinical experience was not significantly associated with familiarity after adjusting for other covariates. Regarding hospital tier, only university-affiliated tertiary hospitals showed a significant difference from primary hospitals, with a negative association (aOR 0.50, 95% CI 0.30–0.83; *P* = 0.008), while non-university tertiary and other tertiary hospitals did not differ significantly from the reference group.

The results also revealed that hospital tier was no longer significantly associated with knowledge of either first-line fluid or first-line vasopressor selection after adjusting for other covariates. This contrasts with the univariate findings, where tertiary hospital setting appeared to confer an advantage, and confirms that the apparent institutional effect was confounded by individual-level factors. For first-line fluid, years of clinical experience emerged as the dominant predictor (overall *P* = 0.015). And professional rank showed a borderline overall association (*P* = 0.058), with both associate senior (aOR: 2.39; 95% CI: 1.02–5.65; *P* = 0.046) and senior ranks (aOR: 2.86; 95% CI: 1.07–7.64; *P* = 0.037) demonstrating significantly higher odds of consistent knowledge compared to junior anesthesiologists. In contrast, academic degree and case volume were not significantly associated with fluid knowledge. For first-line vasopressor, a different set of predictors emerged. Academic degree was significantly associated with consistent knowledge (overall *P* = 0.016). Annual case volume also demonstrated a significant overall association (overall *P* = 0.039). However, neither years of experience nor professional rank showed significant associations with vasopressor knowledge.

For two key outcomes—knowledge of the target MAP and self-reported adherence to guidelines—no variables remained significant in the multivariable models (all *P* > 0.05).

## Discussion

This cross-sectional survey revealed that while most Chinese anesthesiologists reported general awareness of SSC fluid resuscitation recommendations, many demonstrated specific knowledge gaps and practices misaligned with current guidelines. Only 35.6% reported being “very” or “somewhat” familiar with the guidelines, and 72.2% reported compliance—below the 90% benchmark for good compliance [9]. This self-reported rate likely overestimates actual adherence, as evidenced by a multinational survey of Asian ICUs showing compliance rates of only 7.6% for resuscitation bundles and 3.5% for management bundles, far below surveyed rates [10].

This overestimation extends beyond reported compliance to self-perceived familiarity. Indeed in our study, a notable discordance between perceived and actual knowledge emerged among respondents who considered themselves “familiar” with the guidelines. Over one-third failed to identify 65 mmHg as the target MAP, and approximately one-tenth did not recognize balanced crystalloids as first-line fluid. Strikingly, among all respondents who reported familiarity with the guidelines, the majority cited institutional barriers-fluid availability (87.2%) and department convention (74.4%)-as factors influencing their fluid choices. Taken together, these findings suggest that self-perceived familiarity may overestimate true guideline competence, and that system-level factors may override evidence-based practice even among clinicians who report being knowledgeable. This distinction between perceived and actual knowledge is an important consideration when interpreting survey-based studies on clinical knowledge.

The use of vasoactive drugs is a critical component of sepsis management. Early and exclusive use of norepinephrine in septic shock can stabilize MAP and improve survival outcomes [11]. Norepinephrine was first recommended as the vasopressor of choice in the 2008 SSC guidelines [12]. In our study, 86.4% of anesthesiologists selected norepinephrine as their first-line vasopressor. Another survey evaluating adherence to the 2016 SSC guidelines among intensive care practitioners revealed that 96.5% selected norepinephrine as the first-line vasoactive drug, higher than the rate than observed in our study [13]. Regarding hemodynamic monitoring, 77.4% used MAP to guide therapy, consistent with prior studies [14, 15], while dynamic parameters (PPV/SVV) and CRT remained underutilized (6% for CRT) [16, 17]-highlighting opportunities for practical bedside training.

After adjusting for individual characteristics, our study found that hospital tier had no independent effect on either guideline adherence or knowledge. The apparent advantages of tertiary hospitals in univariate analyses were explained by concentration of experienced, higher-ranked clinicians—not institutional factors per se. Multivariate analysis revealed self-reported familiarity was independently associated with professional rank, academic degree, and annual sepsis case volume, with doctoral holders showing 7-fold higher odds. Knowledge of fluid selection was driven by clinical experience and professional rank, whereas vasopressor knowledge associated with academic degree and case volume.

An unexpected finding was that university-affiliated tertiary hospital anesthesiologists reported lower familiarity than primary hospital counterparts. We propose several interrelated explanations. First, response shift bias. Clinicians in academic tertiary centers may apply higher internal standards when rating their own knowledge, potentially leading to more conservative self-assessments compared with their counterparts in non-tertiary settings. Second, clinical exposure and specialization. Anesthesiologists in tertiary hospitals often focus on specific subspecialties (e.g., cardiac or neurosurgical anesthesia), which may limit their routine exposure to general sepsis cases. In addition, higher clinical workloads in tertiary centers may restrict opportunities for continuing education. Third, sampling considerations. The snowball sampling method may have captured different demographic or professional profiles across hospital levels, which could also contribute to the observed differences. Given the unexpected nature of these findings, they should be interpreted cautiously and are presented as hypothesis-generating, warranting further investigation.

These findings carry important implications. First, our findings may inform the design of targeted educational interventions which could be stratified: reinforcing evidence updates for senior clinicians (to potentially reduce experience-dependence), supplementing pharmacological foundations for lower-degree groups, and embedding standardized protocols into high-volume clinical scenarios—rather than merely increasing general awareness. Second, the absence of predictors for self-reported adherence suggests that knowledge alone is likely insufficient. Accordingly, system-level changes addressing resource constraints and departmental culture may be essential to facilitate genuine behavioral change.

This study had several limitations. First, our snowball sampling strategy via WeChat carries inherent limitations, including the absence of a defined sampling frame and the inability to calculate a true response rate. This method may have overrepresented anesthesiologists who are more engaged with academic content or professional social media, potentially introducing selection bias. Therefore, our findings may not be generalizable to the entire population of Chinese anesthesiologists, particularly those with limited academic engagement or restricted access to digital platforms. Second, self-reported familiarity and adherence may not reflect actual clinical behavior; the discordance we observed between perceived and actual knowledge supports this concern. Third, the survey instrument was developed specifically for this study and has not undergone formal psychometric validation. Although pilot testing was conducted, the absence of established reliability and validity metrics may affect measurement precision. Fourth, the survey focused specifically on fluid resuscitation and did not assess other SSC recommendations, limiting the scope of our conclusions. Finally, unmeasured confounders such as individual hospital protocols and resource availability may influence practice patterns.

## Conclusions

This nationwide cross-sectional survey suggests that while more than half of Chinese anesthesiologists reported general familiarity with and adherence to the SSC guidelines, many lacked updated knowledge of specific 2021 recommendations. Individual-level factors were associated with guideline familiarity and knowledge, whereas institutional characteristics appeared to play a limited role after adjustment. These findings may help identify gaps and inform targeted educational interventions for junior anesthesiologists and those with lower academic qualifications, though causal inferences are precluded by the observational design.

## Supplementary Information

Supplementary Material 1.

Supplementary Material 2.

## Data Availability

The anonymised dataset supporting the conclusions of this article is available from the corresponding author on reasonable request.

## References

[1] Singer M, Deutschman CS, Seymour CW, Shankar-Hari M, Annane D, Bauer M, et al. The Third International Consensus Definitions for Sepsis and Septic Shock (Sepsis-3). JAMA. 2016;315(8):801–10. 26903338 10.1001/jama.2016.0287 PMC4968574

[2] Zampieri FG, Bagshaw SM, Semler MW. Fluid Therapy for Critically Ill Adults With Sepsis: A Review. JAMA. 2023;329(22):1967–80. 37314271 10.1001/jama.2023.7560

[3] Dellinger RP, Carlet JM, Masur H, Gerlach H, Calandra T, Cohen J, et al. Surviving Sepsis Campaign guidelines for management of severe sepsis and septic shock. Crit Care Med. 2004;32(3):858–73. 15090974 10.1097/01.ccm.0000117317.18092.e4

[4] Evans L, Rhodes A, Alhazzani W, Antonelli M, Coopersmith CM, French C, et al. Surviving sepsis campaign: international guidelines for management of sepsis and septic shock 2021. Intensive Care Med. 2021;47(11):1181–247. 34599691 10.1007/s00134-021-06506-y PMC8486643

[5] Djurkovic S, Baracaldo JC, Guerra JA, Sartorius J, Haupt MT. A survey of clinicians addressing the approach to the management of severe sepsis and septic shock in the United States. J Crit Care. 2010;25(4):e6581–6. 10.1016/j.jcrc.2010.04.005 20646900

[6] Watkins RR, Haller N, Wayde M, Armitage KB. A Multicenter Survey of House Staff Knowledge About Sepsis and the Surviving Sepsis Campaign Guidelines for Management of Severe Sepsis and Septic Shock. J Intensive Care Med. 2020;35(2):187–90. 29088995 10.1177/0885066617737304

[7] Kahn JM, Davis BS, Yabes JG, Chang CH, Chong DH, Hershey TB, et al. Association Between State-Mandated Protocolized Sepsis Care and In-hospital Mortality Among Adults With Sepsis. JAMA. 2019;322(3):240–50. 31310298 10.1001/jama.2019.9021 PMC6635905

[8] You JS, Park YS, Chung SP, Lee HS, Jeon S, Kim WY, et al. Relationship between time of emergency department admission and adherence to the Surviving Sepsis Campaign bundle in patients with septic shock. Crit Care. 2022;26(1):43. 35148797 10.1186/s13054-022-03899-0 PMC8832860

[9] Kalimouttou A, Lerner I, Cheurfa C, Jannot AS, Pirracchio R. Machine-learning-derived sepsis bundle of care. Intensive Care Med. 2023;49(1):26–36. 36446854 10.1007/s00134-022-06928-2

[10] Phua J, Koh Y, Du B, Tang YQ, Divatia JV, Tan CC, et al. Management of severe sepsis in patients admitted to Asian intensive care units: prospective cohort study. BMJ. 2011;342:d3245. 21669950 10.1136/bmj.d3245 PMC3113333

[11] Morimatsu H, Singh K, Uchino S, Bellomo R, Hart G. Early and exclusive use of norepinephrine in septic shock. Resuscitation. 2004;62(2):249–54. 15294412 10.1016/j.resuscitation.2004.03.016

[12] Hicks P, Cooper DJ. The Surviving Sepsis Campaign: International guidelines for management of severe sepsis and septic shock: 2008. Crit Care Resusc. 2008;10(1):8. 18304010

[13] Bitton E, Zimmerman S, Azevedo LCP, Benhamou D, Cecconi M, De Waele JJ, et al. An international survey of adherence to Surviving Sepsis Campaign Guidelines 2016 regarding fluid resuscitation and vasopressors in the initial management of septic shock. J Crit Care. 2022;68:144–54. 34895959 10.1016/j.jcrc.2021.11.016

[14] Jensen ME, Jensen AS, Meilandt C, Jørgensen KW, Væggemose U, Bach A, et al. Prehospital fluid therapy in patients with suspected infection: a survey of ambulance personnel’s practice. Scand J Trauma Resusc Emerg Med. 2022;30(1):38. 35642066 10.1186/s13049-022-01025-1 PMC9158174

[15] Jessen MK, Simonsen BY, Thomsen MH, Andersen LW, Kolsen-Petersen JA, Kirkegaard H. Fluid management of emergency department patients with sepsis-A survey of fluid resuscitation practices. Acta Anaesthesiol Scand. 2022;66(10):1237–46. 36054552 10.1111/aas.14141 PMC9805143

[16] Hernández G, Ospina-Tascón GA, Damiani LP, Estenssoro E, Dubin A, Hurtado J, et al. Effect of a Resuscitation Strategy Targeting Peripheral Perfusion Status vs Serum Lactate Levels on 28-Day Mortality Among Patients With Septic Shock: The ANDROMEDA-SHOCK Randomized Clinical Trial. JAMA. 2019;321(7):654–64. 30772908 10.1001/jama.2019.0071 PMC6439620

[17] Hernandez G, Carmona P, Ait-Oufella H. Monitoring capillary refill time in septic shock. Intensive Care Med. 2024;50(4):580–2. 38498167 10.1007/s00134-024-07361-3

