# Supplementary material for: A cross-sectional survey of Chinese anesthesiologists’ understanding of fluid resuscitation recommendations in the 2021 surviving sepsis campaign guidelines
Source: BMC Anesthesiol. 2026 Jun 5;26:507. doi: 10.1186/s12871-026-03990-x (PMC13491661; doi:10.1186/s12871-026-03990-x)
Supplement: Supplementary file 1 [file 12871_2026_3990_MOESM1_ESM.pdf]

STROBE Statement—Checklist of items that should be included in reports of ***cross-sectional studies***

	Item No	Recommendation	Reported?	Page/Line
**Title and abstract**	1	(*a*) Indicate the study's design with a commonly used term in the title or the abstract	√	Page 1, lines 1-3
		(*b*) Provide in the abstract an informative and balanced summary of what was done and what was found	√	Page 2-3, abstract section
**Introduction**				
Background/rationale	2	Explain the scientific background and rationale for the investigation being reported	√	Page 3, line 10-22
Objectives	3	State specific objectives, including any prespecified hypotheses	√	Page 4, line 11-12
**Methods**				
Study design	4	Present key elements of study design early in the paper	√	Page 4, line 16-18
Setting	5	Describe the setting, locations, and relevant dates, including periods of recruitment, exposure, follow-up, and data collection	√	Page 4-6 (Methods 2.1, 2.2)
Participants	6	(*a*) Give the eligibility criteria, and the sources and methods of selection of participants	√	Page 5, line 16-17, line 20-22; Page 5, line 1-2
Variables	7	Clearly define all outcomes, exposures, predictors, potential confounders, and effect modifiers. Give diagnostic criteria, if applicable	√	Page 5, line 6-15
Data sources/ measurement	8*	For each variable of interest, give sources of data and details of methods of assessment (measurement). Describe comparability of assessment methods if there is more than one group	√	Page 6, line 3-22
Bias	9	Describe any efforts to address potential sources of bias	√	Page 6, line 3-5; Page 6, 2.3. Non-response bias assessment
Study size	10	Explain how the study size was arrived at	√	Page 5, line 20-22 and Page 6, line 1-2
Quantitative variables	11	Explain how quantitative variables were handled in the analyses. If applicable, describe which groupings were chosen and why	√	Page 7, line 3-7
Statistical methods	12	(*a*) Describe all statistical methods, including those used to control for confounding	√	Page 7: 2.4. Statistical analysis
		(*b*) Describe any methods used to examine subgroups and interactions	√	Page 7, line 8-16
		(*c*) Explain how missing data were addressed	√	Page 5, line 17
		(*d*) If applicable, describe analytical methods taking account of sampling strategy	NA	NA
		(*e*) Describe any sensitivity analyses	√	Page 6, line 13-22
**Results**				
Participants	13*	(a) Report numbers of individuals at each stage of study—eg numbers potentially eligible, examined	√	Page 7-8, Results 3.1. Basic characteristics

		for eligibility, confirmed eligible, included in the study, completing follow-up, and analysed		
		(b) Give reasons for non-participation at each stage	√	Page 5, line 17
		(c) Consider use of a flow diagram	√	Not provided (but numbers clearly stated in text)
Descriptive data	14*	(a) Give characteristics of study participants (eg demographic, clinical, social) and information on exposures and potential confounders	√	Page 7, Page 10 and Page 13
		(b) Indicate number of participants with missing data for each variable of interest	√	Exclusion criteria: >50% missing data but no participants with incomplete responses
Outcome data	15*	Report numbers of outcome events or summary measures	√	Page 7-10 (Results 3.2-3.5)
Main results	16	(*a*) Give unadjusted estimates and, if applicable, confounder-adjusted estimates and their precision (eg, 95% confidence interval). Make clear which confounders were adjusted for and why they were included	√	Page 13 (Results 3.7 and table 2)
		(*b*) Report category boundaries when continuous variables were categorized	√	Page 14 (table 2)
		(*c*) If relevant, consider translating estimates of relative risk into absolute risk for a meaningful time period	NA	NA
Other analyses	17	Report other analyses done—eg analyses of subgroups and interactions, and sensitivity analyses	√	Page 10 (Results 3.6 and table 1); Page 6 (Methods 2.3)
**Discussion**				
Key results	18	Summarise key results with reference to study objectives	√	Page 15, line 4-11
Limitations	19	Discuss limitations of the study, taking into account sources of potential bias or imprecision. Discuss both direction and magnitude of any potential bias	√	Page 18-19
Interpretation	20	Give a cautious overall interpretation of results considering objectives, limitations, multiplicity of analyses, results from similar studies, and other relevant evidence	√	Page 19, line 8-15
Generalisability	21	Discuss the generalisability (external validity) of the study results	√	Page 18, line 12-19
**Other information**				
Funding	22	Give the source of funding and the role of the funders for the present study and, if applicable, for the original study on which the present article is based	√	Page 21, line 4-5

*Give information separately for exposed and unexposed groups.
