# Supplementary material for: A cross-sectional survey of Chinese anesthesiologists’ understanding of fluid resuscitation recommendations in the 2021 surviving sepsis campaign guidelines
Source: BMC Anesthesiol. 2026 Jun 5;26:507. doi: 10.1186/s12871-026-03990-x (PMC13491661; doi:10.1186/s12871-026-03990-x)
Supplement: Supplementary file 2 [file 12871_2026_3990_MOESM2_ESM.pdf]

Introduction

The Surviving Sepsis Campaign (SSC) is a joint initiative of the Society of Critical Care Medicine and the European Society of Intensive Care Medicine. Its goal is to reduce the global incidence and mortality of sepsis and septic shock. The fifth edition of the evidence-based SSC guidelines was published in 2021. Fluid therapy is the cornerstone of hemodynamic resuscitation. As anesthesiologists, keeping up-to-date with sepsis knowledge will improve patient outcomes. This study aims to evaluate anesthesiologists' understanding of fluid therapy recommended by the SSC guidelines.

Please read this introduction and then completion of the questionnaire was taken as voluntary consent to participate and disclose the requested information. All the collected data was kept confidential, and personal identification was not stated in the questionnaire.

Section 1. Demographics

1. **Province where you work: ______________________**
2. **Are you a licensed anesthesiologist?**
   ☐ Yes ☐ No
3. **Type of hospital:**
   ☐ Tertiary-A, university-affiliated
   ☐ Tertiary-A, non-university
   ☐ Other tertiary
   ☐ Secondary or below
4. **Number of operating rooms in your hospital:**
   ☐ <10 ☐ 10–20 ☐ 20–30 ☐ >30
5. **Estimated annual emergency surgical cases with septic shock in your hospital:**
   ☐ <50 ☐ 50–150 ☐ 150–300 ☐ >300
6. **Your highest degree:**
   ☐ Doctorate ☐ Master ☐ Bachelor ☐ Diploma
7. **Years of practice in anesthesia:**
   ☐ <3 ☐ 3–5 ☐ 5–10 ☐ >10
8. **Current professional title:**
   ☐ Chief ☐ Associate Chief ☐ Attending ☐ Resident
9. **Completed standardized residency training?**
   ☐ Yes ☐ No
10. **Duration of residency training (if completed):**
   ☐ 1 yr ☐ 2 yr ☐ 3 yr ☐ 5 yr

Section 2. Professional Knowledge

1. **Primary research interest (choose one):**
   ☐ General anesthesia mechanisms ☐ Pain research ☐ Organ protection ☐ Sepsis ☐ Other _________
2. **Familiarity with the 2021 SSC guidelines on fluid resuscitation:**
   ☐ Very familiar ☐ Somewhat familiar ☐ Slightly familiar ☐ Unaware
3. **Do you follow the 2021 SSC fluid strategy when managing peri-operative septic**

shock?

☐ Yes ☐ No

4. **Preferred fluid strategy in adult sepsis/septic shock:**

   ☐ Early Goal-Directed Therapy (EGDT)

   ☐ Initial bolus + clinical/static parameters

   ☐ Start fluids + dynamic reassessment

5. **Recommended first-line resuscitation fluid in adult sepsis/septic shock:**

   ☐ Crystalloids ☐ Synthetic colloids (gelatin/HES) ☐ Albumin ☐ Plasma

6. **NOT recommended for resuscitation in adult sepsis/septic shock:**

   ☐ Crystalloids ☐ Synthetic colloids (gelatin/HES) ☐ Albumin ☐ Plasma

7. **In patients receiving large-volume crystalloids, add-on recommended:**

   ☐ Crystalloids ☐ Synthetic colloids ☐ Albumin ☐ Plasma

8. **Initial MAP target for septic shock on vasopressors (2021 SSC):**

   ☐ 60 mmHg ☐ 65 mmHg ☐ 70 mmHg ☐ 75 mmHg ☐ 80 mmHg

9. **Time window for broad-spectrum antibiotics in suspected septic shock (1-h bundle):**

   ☐ 1 h ☐ 3 h ☐ 6 h ☐ 12 h ☐ 24 h

10. **When advanced monitoring unavailable, 2021 SSC suggests using __ as additional perfusion index:**

    ☐ Blood pressure ☐ Urine output ☐ Lactate level ☐ Capillary refill time

11. **Select the 3 fluids you most commonly use in elective surgery:**

    ☐ Gelatin ☐ Albumin ☐ 6 % HES ☐ 0.9 % saline ☐ Lactated Ringer's ☐ Acetated Ringer's ☐ Plasma ☐ Whole blood ☐ PRBC

12. **Do you think fluid choices differ between elective surgery and septic shock?**

    ☐ Yes ☐ No

13. **Select the 3 fluids you most commonly use in septic-shock patients on vasopressors:**

    ☐ Gelatin ☐ Albumin ☐ 6 % HES ☐ 0.9 % saline ☐ Lactated Ringer's ☐ Acetated Ringer's ☐ Plasma ☐ Whole blood ☐ PRBC

14. **Top 3 factors influencing your fluid choice (choose 3):**

    ☐ Cost ☐ Commercial promotion ☐ Availability ☐ Department habit ☐ Guideline recommendation ☐ Surgeon preference ☐ Other _________

15. **Top 3 variables you use to guide fluid therapy in septic shock (choose 3):**

    ☐ Urine output ☐ Lactate ☐ MAP ☐ CVP ☐ $SvO_2/ScvO_2$ ☐ SV/SVV/PPV ☐ Echocardiography ☐ Capillary refill time

16. **Reference ranges you consider correct (tick one per item):**

    a) Urine output ≥ ___ mL/kg/h: ☐ 0.2 ☐ 0.5 ☐ 0.8 ☐ 1

    b) Blood lactate ≤ ___ mmol/L: ☐ 0.5 ☐ 1 ☐ 2 ☐ 3 ☐ 5

    c) $SvO_2/ScvO_2$ ≥ ___ %: ☐ 55/60 ☐ 60/65 ☐ 65/70 ☐ 70/75 ☐ 75/80

    d) SVV < ___ %: ☐ 13 ☐ 23 ☐ 33 ☐ 43

    e) PPV ___ %: ☐ 0–10 ☐ 10–20 ☐ 20–30 ☐ 30–40

17. **When selecting monitoring variables, the 3 most important factors are:**

    ☐ Ease of measurement ☐ Senior doctor's order ☐ Department routine ☐ Guideline recommendation ☐ Personal experience ☐ Other _________

18. **First-line vasoactive agent in septic shock (2021 SSC):**
☐ Dopamine ☐ Norepinephrine ☐ Epinephrine ☐ Vasopressin ☐ Dobutamine
19. **Applicability of 2021 SSC fluid resuscitation recommendations to peri-operative anesthesia practice (1 = very inapplicable, 5 = very applicable):**
☐ 1 ☐ 2 ☐ 3 ☐ 4 ☐ 5

Thank you for your participation!
